# Specific MicroRNA Pattern in Colon Tissue of Young Children with Eosinophilic Colitis

**DOI:** 10.3390/ijms18051050

**Published:** 2017-05-12

**Authors:** Zoltán Kiss, Nóra Judit Béres, Erna Sziksz, Bálint Tél, Katalin Borka, András Arató, Attila J. Szabó, Gábor Veres

**Affiliations:** 1 1st Department of Pediatrics, Semmelweis University, Budapest H-1083, Hungary; zoltan.kiss.bio@gmail.com (Z.K.); bnora1988@gmail.com (N.J.B.); szikszerna@gmail.com (E.S.); tel.balint@gmail.com (B.T.); arato.andras@med.semmelweis-univ.hu (A.A.); szabo.attila@med.semmelweis-univ.hu (A.J.S.); 2 MTA-SE Pediatrics and Nephrology Research Group, Budapest H-1083, Hungary; 3 2nd Department of Pathology, Semmelweis University, Budapest H-1091, Hungary; borka.katalin@med.semmelweis-univ.hu

**Keywords:** eosinophilic colitis, microRNA, haematochezia

## Abstract

Eosinophilic colitis (EC) is a common cause of haematochezia in infants and young children. The exact pathomechanism is not understood, and the diagnosis is challenging. The role of microRNAs as key class of regulators of mRNA expression and translation in patients with EC has not been explored. Therefore, the aim of the present study was to explore the miRNA profile in EC with respect to eosinophilic inflammation. Patients enrolled in the study (*n* = 10) had persistent rectal bleeding, and did not respond to elimination dietary treatment. High-throughput microRNA sequencing was carried out on colonic biopsy specimens of children with EC (EC: *n* = 4) and controls (C: *n* = 4) as a preliminary screening of the miRNA profile. Based on the next-generation sequencing (NGS) results and literature data, a potentially relevant panel of miRNAs were selected for further measurements by real-time reverse transcription (RT)-PCR (EC: *n* = 14, C: *n* = 10). Validation by RT-PCR resulted in significantly altered expression of miR-21, -31, -99b, -125a, -146a, -184, -221, -223, and -559 compared to controls (*p* ≤ 0.05). Elevation in miR-21, -99b, -146a, -221, and -223 showed statistically significant correlation to the extent of tissue eosinophilia. Based on our results, we conclude that the dysregulated miRNAs have a potential role in the regulation of apoptosis by targeting Protein kinase B/Mechanistic target of rapamycin (AKT/mTOR)-related pathways in inflammation by modulating Nuclear factor kappa-light-chain-enhancer of activated B cells (NF-κB)-related signalling and eosinophil cell recruitment and activation, mainly by regulating the expression of the chemoattractant eotaxin and the adhesion molecule CD44. Our results could serve as a basis for further extended research exploring the pathomechanism of EC.

## 1. Introduction

Eosinophilic colitis (EC) is a frequent cause of lower gastrointestinal bleeding in the early years of life. The main clinical manifestations of EC are diarrhoea, and the presence of small amounts of mucus and fresh blood in the faeces of otherwise healthy infants [1]. The endoscopic features are generally intact macroscopic structures with swollen erythematous lymphoid follicles (LNH), patchy granularity, and aphthous lesions. Histologically, EC is characterized with eosinophil infiltrate [2,3]. The disease affects both breast-fed and soy or cow’s milk-based formula-receiving children. It has been suggested that the underlying cause could be a non-IgE-mediated hypersensitivity against unidentified dietary antigens in the maternal diet [4]. The most recent study addressing this condition postulated that the recruitment of eosinophils in the colonic mucosa is affected by the expression of eotaxin-2, and degranulation of the perineural mast cells are frequent in EC; however, the exact pathomechanism of EC is unknown, and non-invasive diagnostic tests currently are not available [5,6,7,8,9]. Recently there has been an exponential rise in EC recognition, with an estimated prevalence of 3.3/100,000 [9].

Therapy of EC is based mostly on dietary alterations in the maternal diet or exclusive administration of amino acid-based formulas. Our study focused on patients unresponsive to dietary intervention therapy (both maternal diet restrictions and formula feeding). In these types of cases, persistent bleeding, weight loss, iron deficiency, and anaemia with thrombocytosis can occur; therefore, endoscopy can be considered. However, due to the overlapping symptoms of EC with Crohn’s disease (CD) and several other inflammatory conditions or physical damages of the colon, it is difficult to establish the final diagnosis [10,11,12,13]. This unique and clinically more relevant patient group were previously characterized in the context of endoscopic mucosal histology [10]. Our recent study is a continuation of efforts to investigate the characteristics of these patients.

Regarding the genetic background of EC patients, there are only very limited data available—mostly due to the lack of appropriate tissue specimens [14,15]. However, epigenetic factors gained more importance in recent years, also exerting remarkable effects in diseases with less significant genetic exposure. In some cases, these epigenetic regulators are more closely related to the phenotype; therefore, they have been proposed as suitable biomarkers [16,17,18].

One of the most evolving research areas in epigenetics is that of microRNAs (miRs), the small (20–22 bp) noncoding sequences capable of repressing mRNA translation and affecting biological processes at mRNA and protein level [19]. An increasing number of studies report specific expression alterations of miRs in different gastrointestinal diseases, including eosinophil-associated disorders such as airway allergies or eosinophilic esophagitis (EOE) [16,17,20,21]. However, to the best of our knowledge, data regarding the involvement of miRs in the pathomechanism of EC has not yet been published [20,21,22].

**Figure 1:**
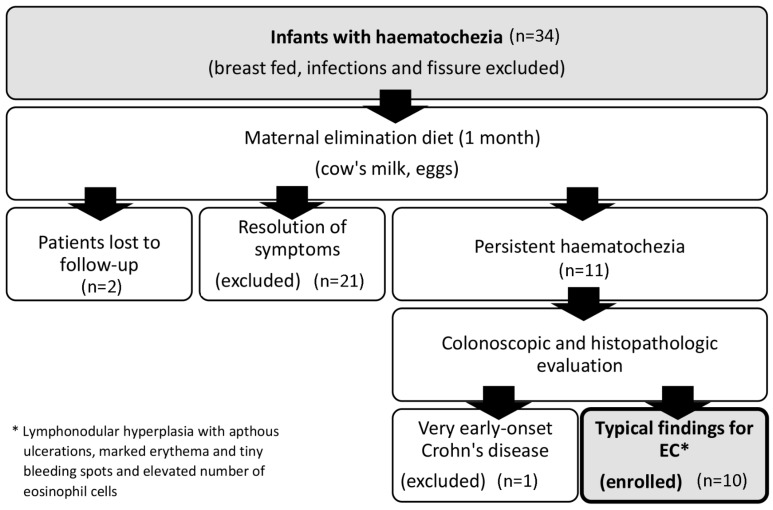
Patient selection chart. EC: eosinophilic colitis.

Therefore, the major aim of our present study was to identify disease-specific miR expression in a group of patients with persistent haematochezia after maternal exclusion diet (Figure 1). Differences may contribute to the diagnostic confirmation of EC in cases with poor progression. Moreover, we aimed to investigate the pool of potential target genes of the altered miRs to gain insight into the pathomechanism of EC.

## 2. Results

### 2.1. EC-Specific miR Expression Profile by Next-Generation Sequencing (NGS)

In the EC biopsies, 456 miRs were dysregulated in some extent, of which 79 miRs had a fold change ≥1.5 in EC patients compared to controls (*p* ≤ 0.05). Seventy-one of these miRs were upregulated, and eight miRs showed lower expression compared to the controls (Table S1). Based on the results of NGS, but with molecular biological relevance as a higher priority, nine dysregulated miRs previously connected to tissue eosinophilia, eosinophil cell-related signalling apoptosis, and inflammation by previous studies were selected for method validation and further analysis by real-time reverse transcription (RT)-PCR [20,21,23,24,25,26,27,28,29,30,31,32].

### 2.2. Expression of miRs Selected for Further Analysis by RT-PCR

**Figure 2:**
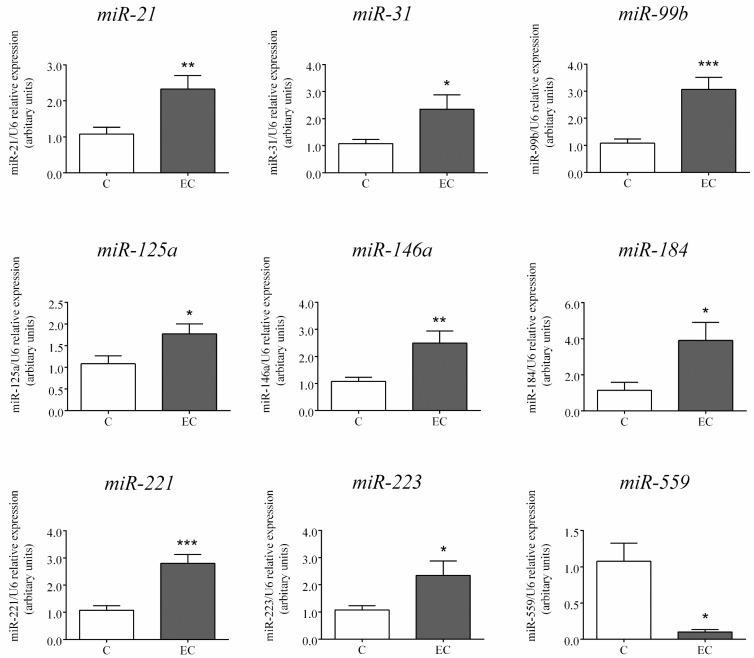
Expression of selected miRs in the colonic mucosa of children with eosinophilic colitis (EC) and controls (C). MiRs were selected based on the results of next-generation sequencing (NGS), and their colonic expression was validated by real-time reverse transcription polymerase chain reaction (RT-PCR). Data are presented as mean ± SEM. * *p* < 0.05, ** *p* < 0.01, *** *p* < 0.001 vs. C.

Similar to the changes found by NGS, the expression of the nine miRs selected for method validation and further analysis by RT-PCR showed statistically significant differences in the colonic mucosa of paediatric patients with EC compared to controls (*p* ≤ 0.05). The expression of miR-21, -31, -99b, -125a, -146a, -184, -221, and -223 was significantly elevated, and that of miR-559 significantly decreased in the colonic mucosa of children with EC compared to controls (Figure 2).

### 2.3. Correlation of miR Expression with Tissue Eosinophil Counts

**Figure 3:**
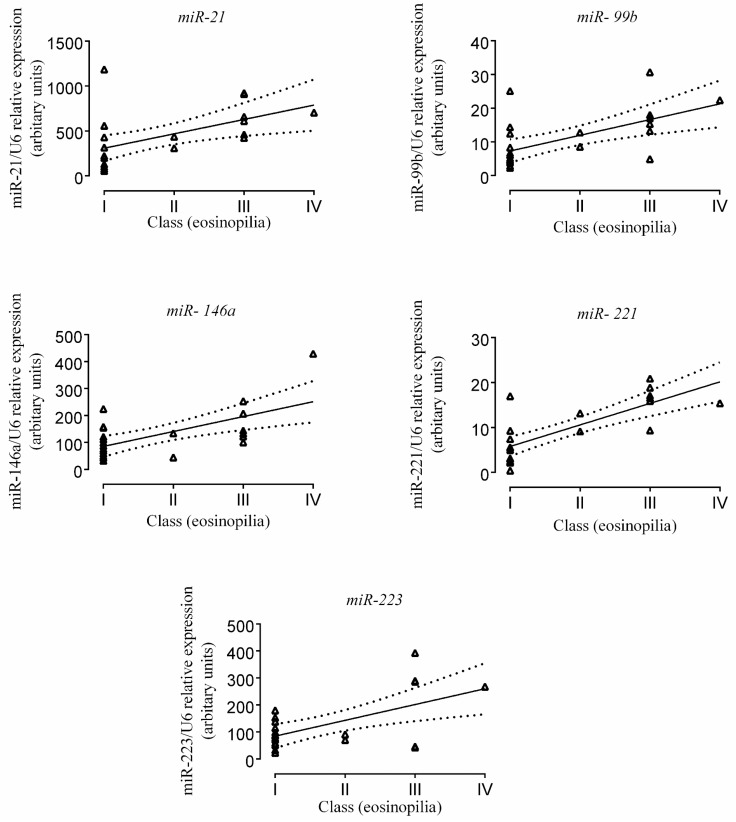
Correlation of miR-21, -99b, -146a, -221, and -223 with the extent of tissue eosinophilia in the colonic mucosa of paediatric patients with eosinophilic colitis. Class I: 0–10 eosinophil/high power field (eo/HPF), Class. II: 10–20 eo/HPF, Class III: 20–40 eo/HPF, Class IV: >40 eo/HPF, area of HPF = 0.2 mm^2^. Solid lines represent a regression line with dotted lines depicting 95% CI.

From the PCR-validated miR panel, expression of miR-21 (*r* = 0.51; 95% CI = 0.15–0.77; *p* = 0.009), -99b (*r* = 0.60; 95% CI = 0.25–0.81, *p* = 0.002), -146a (*r* = 0.62, 95% CI = 0.29–0.82, *p* = 0.001), -221 (*r* = 0.78, 95% CI = 0.78, *p* = 0.53–0.90, *p* < 0.0001) and -223 (*r* = 0.59. 95% CI = 0.22–0.81, *p* = 0.0041) showed a significantly positive correlation with the number of eosinophil granulocytes in paediatric patients with EC. MiR-125a, -31, -184, and 559 showed similar tendency, but the correlations with eosinophil numbers were statistically not significant (Figure 3).

### 2.4. Results of Bioinformatics Analysis

MiRTarBase database contained 386 reporter assay validated targets of the NGS miR profile found in EC. From this list expression of 100 entities were reported in epithelial tissue. Annotation of these 100 entities with gene ontology (GO) terms (biological process domain), and enrichment analysis resulted in 24 GO term categories. The most abundant terms were connected with immune response, regulation of apoptosis, transcription regulation, and cell proliferation (Table S2).

## 3. Discussion

EC in infancy is in most cases a benign self-limiting disease associated with abdominal pain, intestinal dismotility, and mucosal eosinophilia occurring predominantly in the first six months of life [2]. However, several cases with overall poor progression and persistent rectal bleeding were reported (including in our study population). It is of high importance to investigate the pathomechanism and explore the diagnostic potential regarding these particularly sensitive patients. The exact pathomechanism of EC is not understood, and its causative therapy is not available in many cases [10,11,33].

Recently, the role of epigenetic factors came into focus as key determinants in the pathogenesis of different gastrointestinal diseases, including IBD and EOE, frequent forms of eosinophil-associated gastrointestinal disorders (EGID), and bronchial asthma. The expression of several miRs showed a strong correlation with the extent of eosinophil infiltration in the biopsy samples of patients with EOE, and were also dysregulated in their serum [21]. In the current study, we screened the miRNA profile on low patient count in EC with NGS, and based on these preliminary results, we selected a panel of miRNAs strongly connected with eosinophilic inflammation for further analysis. We analysed the selected miRNA with RT-PCR methods on a higher patient number. The panel consisted of nine miRs: miR-21, -31, -99b, -125a, -146a, -184, -221, -223, and -559. Dysregulation of six of these miRs—miR-21, -99b, -125a, -146a, -221, and -223—were previously reported in EOE [20,21]. We selected three additional miRs with strong connection to eosinophil cell recruitment and activation, as described earlier in other conditions. [28,29,32]. The selected miRs also have several potential effects in the modulation of inflammatory pathways not directly affecting eosinophil cells. In the following paragraphs, we will discuss the potential target points, starting with the general pathways of apoptosis followed by the closely relevant molecular events of eosinophilic inflammation.

From the selected miR panel, miR-21, -99b, and -221 are closely related to apoptosis by the potential regulation of the PI3K/Akt/mTOR pathway [34,35,36]. Negative regulation of apoptosis in lymphoid cells and eosinophils could contribute to their accumulation and prolonged survival in the colonic tissue [37]. This mechanism could also be an important factor in tissue infiltration and the subsequent formation of LNH—a frequent endoscopic finding in EC. MiR-559 is an inhibitor of ERBB2 expression. Eosinophil peroxidase—a secreted effector molecule of activated eosinophil cells—is a ligand of ERBB2, and the downstream signalisation originating from ERBB2 has significant proliferative effects; hence, it could contribute to the dwelling caused by prolonged accumulation and activation of eosinophils on inflammatory sites [28].

On the other hand, positive regulation of apoptosis—on which the dysregulated miRs could also have a potential effect according to our present analysis—could slow down the turnover and proliferation of epithelial cells in the colonic tissue, leading to the impairment of intestinal barrier function and the facilitation of inflammatory processes [38,39,40]. This phenomenon as a histologic finding has been reported in connection with EC. Apoptotic cell-specific histochemical TUNEL assay on biopsies labelled with epithelial cell-specific antibodies identified an increased number of apoptotic epithelial cells in the lamina propria of rectosigmoid biopsy specimens originating from infants with haematochezia previously diagnosed with allergic/eosinophilic colitis [40].

**Figure 4:**
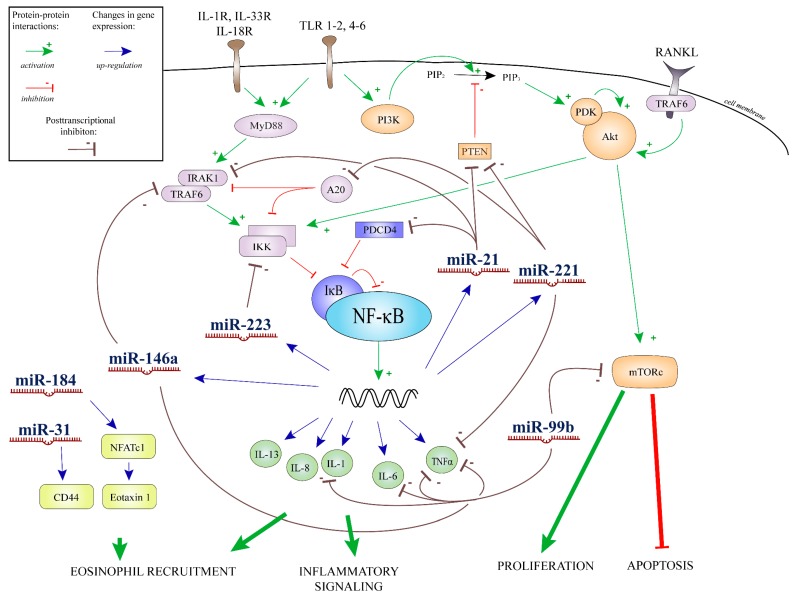
Composite summary graph of the potential target points of the miRs correlated with tissue eosinophil level. Abbreviations: inhibitor of kappa B kinase (IKK), interleukin 1, 6, 8, 13 (IL-1, -6, -8, -13), interleukin receptor-1R, -33R, 18R (IL-1R, -33R, -18R), interleukin-1 receptor-associated kinase 1 (IRAK1), mammalian target of rapamycin complex (mTORc), microRNA-21, -31, -99b, -146a, -184, -221, -223 (miR-21, -31, -99b, -146a, -184, -221, -223), myeloid differentiation primary response 88 (MyD88), nuclear factor kappa-light-chain-enhancer of activated B cells (NF-κB), nuclear factor of activated T-cells, cytoplasmic 1 (NFATc1), nuclear factor of kappa light polypeptide gene enhancer in B cells inhibitor (IκB), phosphatase and tensin homolog (PTEN), phosphatidylinositol (3,4,5)-trisphosphate (PIP3), phosphatidylinositol 4,5-bisphosphate (PIP2), phosphatidylinositol-3-kinase (PI3K), phosphoinositide dependent protein kinase (PDK), programmed cell death protein 4 (PDCD4), protein kinase B (Akt), receptor activator of nuclear factor kappa-B ligand (RANKL), TNF receptor associated factor-6 (TRAF6), Toll-like receptor-1-2, 4-6 (TLR 1-2, 4-6), tumor necrosis factor alpha (TNFα), tumor necrosis factor, α-induced protein 3 (A20).

Similar to the observations in EOE, in the present study we demonstrated significantly elevated expression of miR-21, -146a, and -223 in the colonic mucosa of paediatric patients with EC compared to controls, and it was strongly correlated with the extent of tissue eosinophilia. Furthermore, we found a positive correlation between the expression changes of miR-99b and -221 and the number of tissue eosinophils in the colonic mucosa of EC patients [41,42] (Figure 3). The correlations suggest that these miRs could be actively connected to the pathomechanism of EC. A composite graph of the possible interacting points of these miRs with respect to inflammation and tissue eosinophilia is presented in Figure 4.

Certain dysregulated miRs in the colonic mucosa of children with EC (e.g., miR-125a, -146a, and -223 as identified in our present experiments) are also related to eosinophil cell line differentiation, activation, and recruitment [37]. Lu et al. found a connection between miR-223 deficiency and increased eosinophil progenitor cell proliferation in an ex vivo bone marrow-derived eosinophil culture system [43]. It is inferred from bone marrow culture experiments that miR-125a and -146a are also parts of the regulation network of eosinophil cell maturation [41]. To further investigate the relationship between miRs and eosinophil function in EC, additional studies would be needed on eosinophil granulocytes freshly isolated from the colonic tissues of EC patients.

MiR-21 has been reported to be the most frequently upregulated miR in eosinophil-related diseases [21,23]. It is a complex regulator of eosinophil cell fate, activation and survival. MiR-21 overexpression is able to increase eosinophil progenitor cell growth, it has a pro-survival effect on eosinophils via granulocyte-macrophage colony-stimulating factor (GM-CSF), and acts towards Th2 polarization of the adaptive immune processes [23,25,26,27].

The correlation of miR-221 expression with airway tissue eosinophilia has been reported in a murine model of bronchial asthma. In this model, the level of miR-221 was also correlated with IL-4 secretion, presumably via phosphatase and tensin homolog (PTEN) and nuclear factor kappa-light-chain-enhancer of activated B cells (NF-κB) protein related pathways that are potent inductors of tissue eosinophil infiltration [42]. Increased prevalence of EC has been reported amongst patients with PTEN hamartoma tumour syndromes [44]. MiR-21 and -221 are two miRs which showed significant elevation according to our measurements, and have been connected to the regulation of *PTEN* gene expression [36,45]. PTEN protein is a mon-redundant master regulator of the PI3K signalling pathway. Humans are highly sensitive of PTEN dosage, and alterations in the PI3K/Akt/mTOR pathway has been connected to eosinophil cell-related conditions [46,47,48]. MiR-99b is a member of the miR-99 family, and is also a potent regulator of the PI3K/Akt/mTOR pathway. Besides the effects on eosinophil cell survival, this pathway is active in the tissues of inflammation sites, acting as a mediator of wound healing [35].

MiR-184 targets the NFAT1 transcript directly. Deficiency in the NFAT1 protein product could lead to elevated IL-5, IL-2, and IL-13 production. These cytokines promote eosinophil cell survival and recruit via upregulation of eotaxin levels [28].

MiR-31 levels are positively correlated with the expression of CD44 adhesion molecule, a promoter of tissue eosinophilia. Expression of CD44 in epithelial cells could promote eosinophil migration and activation [31]. It has been demonstrated that changes in CD44 adhesion molecule levels in epithelial cells is a major component of tissue alterations in EOE, and promotes eosinophil and leukocyte migration and activation on the site of inflammation [32].

A limitation of our present study is that biopsies from age-matched healthy subjects could not be obtained. However, we enrolled the youngest possible patients to the control group in order to minimize these effects on the results. To completely eliminate the possible effect of age-related differences, only samples originating from autopsy procedures of infants involved in accidents or who suffered sudden infant death would be completely applicable.

In summary, to the best of our knowledge, we conducted the first experiments in order to define the miR profile specific to EC. Using bioinformatics tools, we assessed the pool of target genes potentially affected by the miRs dysregulated in the colon of children with EC having persistent rectal bleeding, unresponsive to dietary therapies. Our results defined several possible target point for miRs on the molecular biological events of eosinophilic inflammation, and therefore could serve as a base for further investigations of the pathomechanism of EC.

## 4. Materials and Methods

### 4.1. Patients

Samples from all patients enrolled in the study were obtained at the 1st Department of Pediatrics, Semmelweis University, Budapest, Hungary. Colon pinch biopsies were taken from 10 children with EC and 14 controls. Clinical characteristics and laboratory parameters of the patients are shown in Table 1. Patients enrolled in the study had persistent rectal bleeding, and they were unresponsive to dietary intervention therapies. Patients underwent colonoscopy in order to exclude IBD and other organic causes of long-lasting haematochezia. The overall symptoms, long term follow-up, and endoscopic features of the patients did not justify the consideration of monogenic origin. Subsequent diagnosis of CD (without monogenic cause) was obtained in one patient, who was therefore excluded from the study. Diagnosis of EC was based on clinical history, physical examination (no perianal fissure), laboratory findings (infections excluded), endoscopic findings (focal erythema, LNH, and aphta), and pathological evaluation of tissue specimens (average eosinophil count/five high power field (eo/HPF) > 20, HPF area = 0.2 mm^2^) with long-term follow-up of the patients. Microscopic evaluation was open-label conducted by a skilled pathologist as a part of diagnostic processes. Control children underwent colonoscopy to assess the cause of failure to thrive or suspicion of polyp or Meckel-diverticulum. All specimens in the control group showed normal macroscopic and histologic features. Biopsies were immediately snap-frozen and stored at −80 °C until further experiments. Written informed consent was obtained from the parents prior to the procedure, and the study was approved by the Semmelweis University Regional and Institutional Committee for Research Ethics (TUKEB No.: 10408/2012, Date: 30 March 2012).

### 4.2. RNA Isolation

Total RNA isolation from the colonic biopsies was conducted with TRIzol reagent (Ambion, Austin, TX, USA) for the next-generation sequencing (NGS) and Quick-RNA MiniPrep isolation kit (Zymo Research, Irvine, CA, USA) for the real-time reverse transcription polymerase chain reaction (RT-PCR) measurements, according to the manufacturer’s protocol.

### 4.3. cDNA Library Preparation and NGS

A cDNA library for small RNA-Seq was generated from 1 μg total RNA samples derived from the colonic mucosa of paediatric EC patients (EC: *n* = 4) and controls (C: *n* = 4) using TruSeq Small RNA Sample Preparation Kit (Illumina, San Diego, CA, USA) according to the manufacturer’s protocol. Fragment size distribution and molarity of libraries were checked on Agilent BioAnalyzer DNA1000 chip (Agilent Technologies, Santa Clara, CA, USA). Concentration of small RNA libraries were set to 10 nM, and cluster generation was done using TruSeq SR Cluster kit v3-cBot-HS kit on cBot instrument, then a single read 50 bp sequencing run was performed on Illumina HiScan SQ instrument (Illumina, San Diego, CA, USA) carried out by UD-GenoMed Medical Genomic Technologies Ltd. (Debrecen, Hungary).

### 4.4. RT-PCR

RT-PCRs were performed on colonic biopsy samples of EC patients (*n* = 14) and controls (*n* = 10). Total RNA was reverse-transcribed using TaqMan MicroRNA Reverse Transcription Kit (Life Technologies, Carlsbad, CA, USA). MiRs were selected for validation according to the NGS results, and their expression was determined by RT-PCR using TaqMan Universal PCR Master Mix No AmpErase UNG (Life Technologies). Measurements were carried out on a LightCycler 480 instrument (Roche, Basel, Switzerland). Relative expression level was calculated by the ΔΔ*C*q formula, using U6 as an internal standard. TaqMan miR assays (miR-21: ID: 000397, miR-31: ID: 002279, miR-99b: ID: 000436, miR-125a: ID: 002198, miR-146a: ID: 000468, miR-184: ID: 000485, miR-221: ID: 000524, miR-223: ID: 002295, miR-559: ID: 001527, U6: ID: 001973) were used according to the manufacturer’s instructions.

### 4.5. Bioinformatics Analysis of the Whole NGS miR Profile

Experimentally validated (reporter assay) target genes were selected from the MiRTarBase database (Available online: http://mirtarbase.mbc.nctu.edu.tw) [49]. Target genes were filtered for site of expression reported according to the tissue expression database of UniProt (Available online: http://www.uniprot.org) [50]. The Database for Annotation, Visualization and Integrated Discovery (DAVID) bioinformatics tool was used to annotate the resulting list in the Biological process domain of the Gene Ontology (GO) database [51,52,53,54]. Enrichment analysis was carried out on the results with an established 0.01 threshold of the modified Fisher Exact *p*-value (EASE) scores of the enriched categories adjusted with the Benjamini–Hochberg correction method, and 10 entities threshold for group count.

### 4.6. Determination of Eosinophil Counts in the Colonic Tissue of EC Patients

**Figure 5:**
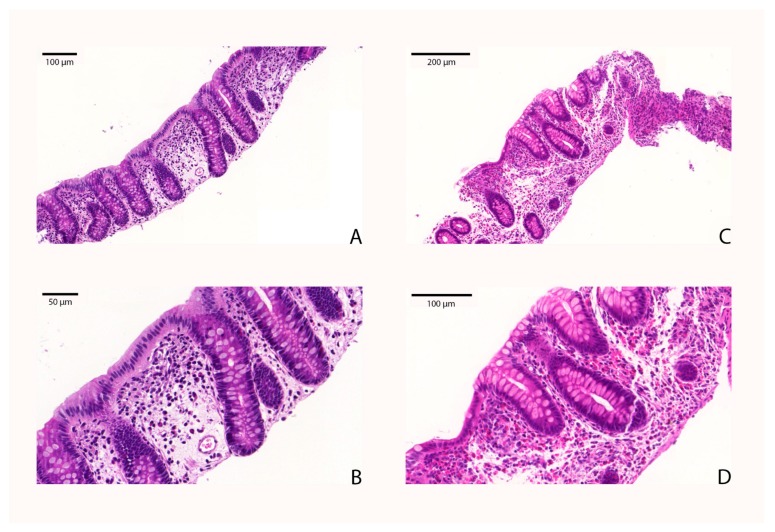
Reference of the histologic assessment. Detection of eosinophil cells with Hematoxylin and Eosin (H&E) staining: 3–4/HPF eosinophil cells were detectable in normal biopsy (**A**, **B**). In a patient with eosinophil colitis, 100–120 eosinophils per HPF are seen (**C**, **D**).

Colonic tissue eosinophil counts were assessed in the microscopic slides by a pathologist with an assumption of representativeness. Evaluation of the specimens was open-label and conducted as a part of the normal diagnostic procedure. Paraffin-embedded tissue samples and fresh-frozen specimens were taken within close vicinity of each other. Eosinophil cells were detected by standard Hematoxylin and Eosin (H&E) staining (ST5020 Multistainer, Leica Microsystems GmbH, Wetzlar, Germany). Eosinophils in the lamina propria were counted in five random high-power fields (HPF: area of tissue covered by a ×40 light microscope objective: 0.2 mm^2^) selected from an eosinophil-dense part of the slides, on optical microscopy (BX51TF, Olympus, Tokyo, Japan). More than 20 eo/HPF was the cut-off number for EC (reference of histological evaluation presented in Figure 5). Based on the average number of eosinophil granulocytes in five HPFs (HPF area = 0.2 mm^2^), patients were divided into four categories (Class I: 0–10 eo/HPF, Class II: 10–20 eo/HPF, Class III: 20–40 eo/HPF, Class IV: >40 eo/HPF).

Correlation between tissue eosinophil counts and miR expressions was calculated using a Spearman method assuming Gaussian distribution of the data.

### 4.7. Statistical Analysis

Statistical analysis was performed using the GraphPad statistical software package (GraphPad Software, La Jolla, CA, USA). Shapiro–Wilk test was used to test normality. Data were analysed by unpaired, two-tailed *t*-test and presented as mean ± standard error of the mean (SEM) and normalized to the control group. Two-tailed *p*-values were estimated in all the statistical calculations, and results were filtered after a 0.05 *p*-value threshold for significance.

## Figures and Tables

**Table 1:** Clinical characteristics and laboratory parameters of patients enrolled in the study (**A**) in the next-generation sequencing groups; and (**B**) in the real-time reverse transcription polymerase chain reaction validation groups. BMI: body mass index, CRP: C-reactive protein; Data are presented as mean ± SD * *p* ≤ 0.05 vs. Control, *** *p* ≤ 0.0001 vs. Control.

**A**	**Control**	**Eosinophilic Colitis**
*n*	4	4
Male/Female	2/2	3/1
Age (months)	136.5 ± 35.78	8.88 ± 5.14 *
BMI (kg/m^2^)	-	18.66 ± 3.79
Iron (µmol/L)	10.75 ± 2.5	13.25 ± 3.57
Albumin (g/L)	51.75 ± 8.81	-
Haemoglobin (g/L)	134.3 ± 7.76	121.8 ± 3.64
Haematocrit (%)	0.39 ± 0.02	0.34 ± 0.013
Platelet count (Giga/L)	325.8 ± 21.10	440.5 ± 57.81
CRP (mg/L)	1.25 ± 1.4	0
**B**	**Control**	**Eosinophilic Colitis**
*n*	14	10
Male/Female	6/8	8/2
Age (months)	29.82 ± 17.08	5.7 ± 4.76 ***
BMI (kg/m^2^)	16.15 ± 1.6	16.21 ± 1.74
Length-weight percentile (%)	-	42.25 ± 34.92 1/10 overweight 1/10 underweight
Iron (µmol/L)	15.70 ± 4.9	9.89 ± 5.62 *
Albumin (g/L)	45.8 ± 2.01	44 ± 4.24
Haemoglobin (g/L)	122.1 ± 12.26	112.6 ± 8.32 *
Haematocrit (%)	35.18 ± 2.8	32 ± 1.94
Platelet count (Giga/L)	364.6 ± 87.09	394.5 ± 104.1
CRP (mg/L)	0.79 ± 1.4	3.44 ± 8.49

## Supplementary Materials

Supplementary materials can be found at www.mdpi.com/1422-0067/18/5/1050/s1.
