# Supplementary material for: Specific MicroRNA Pattern in Colon Tissue of Young Children with Eosinophilic Colitis
Source: Int J Mol Sci. 2017 May 12;18(5):1050. doi: 10.3390/ijms18051050 (PMC5454962; doi:10.3390/ijms18051050)
Supplement: Supplementary file 1 [file ijms-18-01050-s001.zip › Supplementary Table 2.pdf]

Supplementary Table 2. Target genes of miRs resulted from the next generation sequencing of colonic samples and their related gene ontology (GO) terms (.B-H: P- values were corrected by the Benjamini-Hochberg method).

GO Terms	*Genes*	*B-H*
positive regulation of transcription from RNA polymerase II promoter	*E2F1, HMGB1, CCNT1, TLR2, ZEB1, REST, SRF, APP, HNRNPK, IL1B, YAP1, MYC, BCL9, AKT2, EGFR, NTF3, SMAD2, HMGA1, BRCA1, STAT3, UHRF1, EP300, NCOA3, SP1, VEGFA, MAPK3, SMARCA4*	*0.0000*
transcription, DNA-templated	*E2F1, BMI1, ERG, ERBB2, RCOR1, CCNT1, EZH2, YBX3, FASLG, SMAD2, ZEB1, TOPORS, DAXX, BRCA1, PURA, EYA4, UHRF1, TSPYL2, SP1, KHSRP, DNMT1, LRRFIP1, MN1, MYC, HDAC6, SMARCA4*	*0.0046*
negative regulation of transcription from RNA polymerase II promoter	*E2F1, BMI1, DNMT3A, HMGB1, PHB, RCOR1, EZH2, YBX3, FASLG, SMAD2, ZEB1, REST, DAXX, SNAI1, STAT3, UHRF1, EP300, HNRNPK, VEGFA, DNMT1, IL1B, MYC, SMARCA4*	*0.0000*
innate immune response	*EGFR, HMGB1, GRB2, MAP2K3, ERBB2, WASF2, TLR2, IRS1, PTEN, IKBKE, APP, CDKN1A, EP300, CDKN1B, MAPK3, IL1B, SHC1, LRRFIP1, TNFAIP3*	*0.0008*
gene expression	*DNMT3A, YWHAZ, EZH2, CCNT1, ELAVL1, SMAD2, SMN1, UHRF1, EP300, HNRNPK, SP1, PCBP1, WDR77, MAPK3, KHSRP, WDR4, DNMT1, YAP1, MYC*	*0.0021*
signal transduction	*EGFR, RET, YWHAZ, ERG, NTF3, TP53BP2, PHB, MAP2K3, ERBB2, TLR2, FASLG, IRS1, STAT3, ATM, HNRNPK, HGS, IL1B, AKT2*	*0.0109*
negative regulation of transcription, DNA-templated	*E2F1, PHB, RCOR1, EZH2, SMAD2, REST, ZEB1, DAXX, PDCD4, HMGA1, BRCA1, PURA, CDKN1B, PBXIP1, LRRFIP1, HDAC6, SMARCA4*	*0.0000*
apoptotic process	*E2F1, RTN4, HMGB1, YWHAZ, TGFBR2, TLR2, FASLG, DAXX, PDCD4, PTEN, BRCA1, EP300, MAPK3, IL1B, APAF1, TNFAIP3, APC*	*0.0001*
positive regulation of transcription, DNA-templated	*E2F1, RET, MAP2K3, PHB, SMAD2, REST, TOPORS, SNAI1, HMGA1, STAT3, BRCA1, SP1, NCOA3, IL1B, MYC, SMARCA4*	*0.0001*
negative regulation of cell proliferation	*CDKN1A, CDKN1B, INPPL1, PHB, HGS, IL1B, SMAD2, ZEB1, REST, SRF, PTEN, HMGA1, STAT3, APC*	*0.0002*
viral process	*MSH6, GRB2, RCOR1, WASF2, CCNT1, DAXX, HMGA1, STAT3, EP300, HNRNPK, SP1, MAPK3, BNIP3L, SHC1*	*0.0025*
positive regulation of cell proliferation	*EGFR, NTF3, TGFBR2, FASLG, PTEN, IRS1, PURA, CDKN1B, WDR77, VEGFA, SHC1, YAP1, MYC*	*0.0025*
negative regulation of apoptotic process	*EGFR, CDKN1A, YWHAZ, CDKN1B, HNRNPK, CD44, BNIP3L, VEGFA, TOPORS, PTEN, PDCD4, MYC, STAT3*	*0.0036*
blood coagulation	*YWHAZ, BSG, GRB2, RCOR1, L1CAM, WEE1, SLC7A11, APP, CD44, ATP2A2, VEGFA, MAPK3, SHC1*	*0.0036*
aging	*DNMT3A, HNRNPK, TFRC, TGFBR2, DNMT1, IL1B, SHC1, APAF1, CCL5, PTEN, STAT3*	*0.0000*
neurotrophin TRK receptor signaling pathway	*RTN4, EGFR, CDKN1A, CDKN1B, GRB2, ERBB2, MAPK3, SHC1, PTEN, IRS1, STAT3*	*0.0109*
positive regulation of cell migration	*EGFR, RET, NTF3, VEGFA, NUMB, CCL5, IRS1, SNAI1, AKT2, APC*	*0.0002*
positive regulation of gene expression	*E2F1, CD44, NCOA3, VEGFA, TLR2, DNMT1, IL1B, HGS, HSPA1B, BRCA1*	*0.0017*
MAPK cascade	*EGFR, RET, GRB2, ERBB2, MAPK3, IL1B, SHC1, CCL5, IRS1, MYC*	*0.0021*
nervous system development	*BDNF, EP300, NTF3, VEGFA, L1CAM, APAF1, STAT3, SMN1, SMARCA4, PURA*	*0.0035*

epidermal growth factor receptor signaling pathway	*EGFR, CDKN1A, CDKN1B, GRB2, ERBB2, MAPK3, HGS, SHC1, PTEN, IRS1*	*0.0106*
response to drug	*CDKN1A, RET, BDNF, CDKN1B, TGFBR2, CCL5, PTEN, MYC, STAT3, APC*	*0.0111*
regulation of transcription from RNA polymerase II promoter	*HMGB1, ERG, VEGFA, WDR77, LRRFIP1, ZEB1, BRCA1, STAT3, SMARCA4, PURA*	*0.0225*
axon guidance	*EGFR, BDNF, NTF3, GRB2, ERBB2, MAPK3, NUMB, SHC1, L1CAM, IRS1*	*0.2818*
