# Supplementary material for: Specific MicroRNA Pattern in Colon Tissue of Young Children with Eosinophilic Colitis
Source: Int J Mol Sci. 2017 May 12;18(5):1050. doi: 10.3390/ijms18051050 (PMC5454962; doi:10.3390/ijms18051050)
Supplement: Supplementary file 1 [file ijms-18-01050-s001.zip › Supplementary Table 1.pdf]

Supplementary table 1*. List of disregulated microRNAs in the NGS experiment*

microRNA ID	***P-value (EC vs. K)***	***Fold change (EC vs. K)***
hsa-mir-10b	*0.74*	*-1.09*
hsa-mir-192	*0.32*	*-1.69*
hsa-mir-26a-1	*0.01*	*-14.90*
hsa-mir-10a	*0.11*	*1.71*
hsa-mir-143	*0.32*	*-1.51*
hsa-mir-375	*0.35*	*1.88*
hsa-mir-148a	*0.04*	*2.05*
hsa-mir-181a-2	*0.35*	*1.48*
hsa-mir-191	*0.01*	*-13.04*
hsa-mir-21	*0.00*	*3.55*
hsa-mir-378	*0.46*	*1.43*
hsa-mir-27b	*0.01*	*2.77*
hsa-mir-22	*0.04*	*3.97*
hsa-let-7a-1	*0.16*	*-2.52*
hsa-let-7f-2	*0.33*	*-1.96*
hsa-mir-378c	*0.17*	*2.15*
hsa-mir-92a-2	*0.00*	*3.58*
hsa-mir-28	*0.18*	*1.62*
hsa-mir-141	*0.22*	*1.79*
hsa-mir-486	*0.97*	*-1.02*
hsa-let-7i	*0.10*	*1.61*
hsa-mir-142	*0.10*	*2.21*
hsa-let-7g	*0.33*	*-1.47*
hsa-mir-146a	*0.03*	*5.62*
hsa-mir-27a	*0.02*	*2.56*
hsa-mir-186	*0.31*	*1.80*
hsa-mir-30a	*0.28*	*1.56*
hsa-mir-451	*0.05*	*-6.75*
hsa-mir-215	*0.38*	*-1.50*
hsa-mir-127	*0.13*	*-2.28*
hsa-mir-16-2	*0.06*	*3.20*
hsa-let-7b	*0.19*	*-1.47*
hsa-mir-99b	*0.66*	*1.37*
hsa-mir-145	*0.09*	*-3.65*
hsa-mir-25	*0.00*	*2.67*
hsa-mir-103-1-as	*0.07*	*4.78*
hsa-mir-125a	*0.15*	*-2.29*
hsa-mir-126	*0.11*	*-1.61*
hsa-mir-194-2	*0.80*	*-1.14*
hsa-mir-182	*0.14*	*-2.07*
hsa-mir-151	*0.78*	*-1.10*
hsa-mir-101-2	*0.89*	*1.04*

hsa-mir-19b-2	*0.12*	*-2.09*
hsa-mir-30d	*0.02*	*2.07*
hsa-mir-199a-1	*0.36*	*1.74*
hsa-mir-200c	*0.61*	*-1.25*
hsa-mir-221	*0.06*	*2.83*
hsa-mir-199b	*0.20*	*2.28*
hsa-mir-98	*0.72*	*-1.32*
hsa-mir-29a	*0.11*	*3.04*
hsa-mir-320a	*0.18*	*2.46*
hsa-mir-3184	*0.05*	*4.58*
hsa-mir-181b-2	*0.06*	*2.58*
hsa-mir-100	*0.20*	*3.26*
hsa-mir-30b	*0.00*	*-8.64*
hsa-mir-24-1	*0.59*	*1.38*
hsa-mir-26b	*0.85*	*-1.12*
hsa-mir-146b	*0.10*	*-2.65*
hsa-mir-429	*0.08*	*2.66*
hsa-mir-93	*0.06*	*-3.92*
hsa-mir-23a	*0.28*	*1.85*
hsa-mir-532	*0.15*	*1.69*
hsa-mir-30e	*0.18*	*1.83*
hsa-mir-23b	*0.92*	*-1.08*
hsa-mir-200a	*0.10*	*2.13*
hsa-mir-181c	*0.07*	*2.17*
hsa-mir-574	*0.02*	*-3.62*
hsa-mir-203	*0.30*	*1.39*
hsa-mir-155	*0.62*	*-1.49*
hsa-mir-1307	*0.04*	*3.68*
hsa-mir-340	*0.46*	*1.38*
hsa-mir-148b	*0.29*	*1.62*
hsa-mir-769	*0.20*	*-1.67*
hsa-mir-150	*0.23*	*-1.78*
hsa-mir-92b	*0.00*	*5.76*
hsa-mir-106b	*0.24*	*1.44*
hsa-mir-342	*0.54*	*-1.45*
hsa-let-7d	*0.10*	*1.64*
hsa-mir-200b	*0.98*	*1.01*
hsa-mir-425	*0.67*	*1.28*
hsa-mir-195	*0.67*	*-1.20*
hsa-let-7e	*0.20*	*-3.17*
hsa-mir-30c-2	*0.01*	*-5.77*
hsa-mir-23c	*0.59*	*-26.81*
hsa-mir-125b-2	*0.71*	*1.25*
hsa-mir-19a	*0.24*	*-1.72*
hsa-mir-210	*0.20*	*1.57*
hsa-mir-29c	*0.64*	*1.33*

hsa-mir-130a	*0.03*	*5.14*
hsa-mir-941-3	*0.94*	*1.05*
hsa-mir-381	*0.04*	*4.13*
hsa-mir-196b	*0.14*	*-2.41*
hsa-mir-15a	*0.02*	*6.09*
hsa-mir-361	*0.41*	*1.45*
hsa-mir-484	*0.51*	*-1.48*
hsa-mir-107	*0.08*	*3.65*
hsa-mir-409	*0.48*	*1.44*
hsa-mir-197	*0.69*	*-1.23*
hsa-mir-335	*0.25*	*1.45*
hsa-mir-660	*0.04*	*1.83*
hsa-mir-136	*0.30*	*1.77*
hsa-mir-196a-1	*0.89*	*-1.07*
hsa-mir-15b	*0.58*	*-1.25*
hsa-mir-339	*0.82*	*1.14*
hsa-let-7c	*0.06*	*-2.28*
hsa-mir-29b-1	*0.00*	*-7.08*
hsa-mir-374a	*0.24*	*-3.79*
hsa-mir-1248	*0.18*	*-8.14*
hsa-mir-1260	*0.32*	*-3.40*
hsa-mir-20a	*0.46*	*-10.87*
hsa-mir-3120	*0.56*	*-1.46*
hsa-mir-411	*0.28*	*-3.50*
hsa-mir-497	*0.31*	*2.30*
hsa-mir-17	*0.20*	*-4.97*
hsa-mir-1290	*0.05*	*4.18*
hsa-mir-320b-2	*0.01*	*12.57*
hsa-mir-181a-1	*0.30*	*1.46*
hsa-mir-501	*0.51*	*1.65*
hsa-mir-147b	*0.72*	*-1.20*
hsa-mir-1246	*0.11*	*-3.64*
hsa-mir-338	*0.77*	*-1.26*
hsa-mir-183	*0.92*	*1.04*
hsa-mir-362	*0.32*	*-5.12*
hsa-mir-4328	*0.57*	*-5.90*
hsa-mir-130b	*0.00*	*9.82*
hsa-mir-9-2	*0.11*	*-6.40*
hsa-mir-301a	*0.25*	*-7.05*
hsa-mir-132	*0.18*	*1.85*
hsa-mir-324	*0.21*	*-1.98*
hsa-mir-193b	*0.97*	*-1.01*
hsa-mir-500a	*0.29*	*2.10*
hsa-mir-374c	*0.45*	*-1.73*
hsa-mir-345	*0.12*	*2.69*
hsa-mir-424	*0.08*	*2.14*

hsa-mir-128-2	*0.02*	*4.69*
hsa-mir-577	*0.49*	*-1.65*
hsa-mir-744	*0.44*	*-3.92*
hsa-mir-582	*0.37*	*-1.55*
hsa-mir-144	*0.07*	*-1.88*
hsa-mir-654	*0.10*	*-3.19*
hsa-mir-204	*0.77*	*-1.33*
hsa-mir-223	*0.62*	*1.28*
hsa-mir-222	*0.56*	*1.39*
hsa-mir-181d	*0.61*	*-1.27*
hsa-mir-410	*0.03*	*5.82*
hsa-mir-663	*0.00*	*32.23*
hsa-mir-99a	*0.79*	*1.59*
hsa-mir-1260b	*0.56*	*-9.73*
hsa-mir-106a	*0.15*	*-5.04*
hsa-mir-421	*0.00*	*3.94*
hsa-mir-589	*0.19*	*2.58*
hsa-mir-34a	*0.63*	*1.58*
hsa-mir-152	*0.56*	*1.99*
hsa-mir-1296	*0.10*	*-1.97*
hsa-mir-193a	*0.91*	*-1.05*
hsa-mir-652	*0.98*	*-1.02*
hsa-mir-454	*0.36*	*-3.32*
hsa-mir-185	*0.83*	*1.17*
hsa-mir-140	*0.40*	*2.75*
hsa-mir-598	*0.01*	*-2.50*
hsa-mir-619	*0.00*	*31.78*
hsa-mir-134	*0.59*	*-1.26*
hsa-mir-1972-2	*0.00*	*79.29*
hsa-mir-542	*0.08*	*1.83*
hsa-mir-708	*0.13*	*2.63*
hsa-mir-1304	*0.01*	*3.47*
hsa-mir-1261	*0.72*	*-1.48*
hsa-mir-149	*0.41*	*1.57*
hsa-mir-1908	*0.00*	*20.12*
hsa-mir-7-2	*0.35*	*-1.88*
hsa-mir-3185	*0.02*	*9.93*
hsa-mir-432	*0.92*	*-1.04*
hsa-mir-18a	*0.12*	*-14.06*
hsa-mir-664	*0.63*	*1.64*
hsa-mir-331	*0.66*	*-1.36*
hsa-mir-671	*0.61*	*1.38*
hsa-mir-3613	*0.35*	*-1.93*
hsa-mir-363	*0.20*	*4.53*
hsa-mir-328	*0.01*	*7.51*
hsa-mir-7-1	*0.62*	*1.15*

hsa-mir-889	*0.32*	*-3.50*
hsa-mir-3687	*0.00*	*164.32*
hsa-mir-105-2	*0.46*	*2.34*
hsa-mir-32	*0.10*	*2.64*
hsa-mir-139	*0.84*	*-1.09*
hsa-mir-455	*0.76*	*-1.16*
hsa-mir-1247	*0.43*	*4.13*
hsa-mir-452	*0.49*	*1.50*
hsa-mir-212	*0.09*	*-24.50*
hsa-mir-3135	*0.49*	*4.09*
hsa-mir-1237	*0.01*	*65.46*
hsa-mir-133a-2	*0.43*	*-2.26*
hsa-mir-1285-1	*0.02*	*9.42*
hsa-mir-874	*0.15*	*3.31*
hsa-mir-33b	*0.93*	*1.07*
hsa-mir-502	*0.17*	*2.31*
hsa-mir-1208	*0.02*	*54.41*
hsa-mir-3137	*0.24*	*4.54*
hsa-mir-374b	*0.45*	*-1.73*
hsa-mir-369	*0.02*	*6.18*
hsa-mir-548k	*0.97*	*1.05*
hsa-mir-3131	*0.70*	*-1.23*
hsa-mir-592	*0.06*	*3.63*
hsa-mir-218-2	*0.22*	*-6.08*
hsa-mir-552	*0.10*	*2.40*
hsa-mir-505	*0.46*	*2.18*
hsa-mir-718	*0.02*	*25.64*
hsa-mir-3195	*0.52*	*-1.83*
hsa-mir-1180	*0.19*	*2.40*
hsa-mir-493	*0.21*	*-2.54*
hsa-mir-1273	*0.00*	*23.91*
hsa-mir-625	*0.00*	*15.45*
hsa-mir-299	*0.02*	*2.76*
hsa-mir-1275	*0.13*	*-12.22*
hsa-mir-1292	*0.00*	*18.37*
hsa-mir-1281	*0.00*	*15.56*
hsa-mir-629	*0.02*	*4.41*
hsa-mir-138-1	*0.91*	*1.13*
hsa-mir-33a	*0.17*	*-2.03*
hsa-mir-30c-1	*0.40*	*-3.56*
hsa-let-7f-1	*0.08*	*2.00*
hsa-mir-1303	*0.02*	*9.24*
hsa-mir-377	*0.83*	*-1.18*
hsa-mir-382	*0.76*	*1.29*
hsa-mir-3656	*0.00*	*27.13*
hsa-mir-584	*0.01*	*9.33*

hsa-mir-96	*0.50*	*-2.42*
hsa-mir-887	*0.54*	*2.78*
hsa-mir-561	*0.83*	*1.11*
hsa-mir-125b-1	*0.63*	*2.78*
hsa-mir-95	*0.91*	*1.10*
hsa-mir-483	*0.01*	*23.40*
hsa-mir-487b	*0.80*	*-1.18*
hsa-mir-1469	*0.02*	*17.01*
hsa-mir-3614	*0.03*	*3.51*
hsa-mir-651	*0.72*	*1.46*
hsa-mir-135a-1	*0.82*	*1.20*
hsa-mir-3180-3	*0.31*	*1.85*
hsa-mir-1301	*0.64*	*-1.56*
hsa-mir-153-2	*0.33*	*-2.73*
hsa-mir-576	*0.12*	*2.39*
hsa-mir-3607	*0.87*	*1.18*
hsa-mir-365-2	*0.85*	*-1.23*
hsa-mir-4281	*0.00*	*21.48*
hsa-mir-26a-2	*0.83*	*-1.28*
hsa-mir-34c	*0.60*	*-1.74*
hsa-mir-337	*0.30*	*-3.11*
hsa-mir-3141	*0.05*	*3.44*
hsa-mir-3162	*0.78*	*1.30*
hsa-mir-365-1	*0.73*	*2.09*
hsa-mir-548e	*0.03*	*5.04*
hsa-mir-376a-1	*0.36*	*-4.32*
hsa-mir-590	*0.20*	*-7.08*
hsa-mir-596	*0.00*	*58.64*
hsa-mir-638	*0.00*	*33.40*
hsa-mir-370	*0.97*	*1.04*
hsa-mir-4286	*0.67*	*-1.71*
hsa-mir-3692	*0.52*	*-3.28*
hsa-mir-485	*0.38*	*-3.90*
hsa-mir-636	*0.70*	*26.30*
hsa-mir-24-2	*0.94*	*1.07*
hsa-mir-762	*0.62*	*14.04*
hsa-mir-766	*0.49*	*1.96*
hsa-mir-548i-4	*0.42*	*-3.40*
hsa-mir-296	*0.02*	*5.38*
hsa-mir-1249	*0.03*	*3.36*
hsa-mir-3196	*0.01*	*5.91*
hsa-mir-665	*0.45*	*4.31*
hsa-mir-135b	*0.30*	*-4.37*
hsa-mir-330	*0.81*	*1.47*
hsa-mir-450a-2	*0.91*	*1.11*
hsa-mir-101-1	*0.93*	*1.09*

hsa-mir-3936	*0.60*	*-2.98*
hsa-mir-548f-1	*0.99*	*-1.02*
hsa-mir-1273d	*0.02*	*18.04*
hsa-mir-450b	*0.10*	*7.27*
hsa-mir-1181	*0.02*	*5.01*
hsa-mir-663b	*0.71*	*1.49*
hsa-mir-3620	*0.01*	*6.38*
hsa-mir-320c-2	*0.19*	*4.63*
hsa-mir-3142	*0.72*	*19.47*
hsa-mir-548t	*0.01*	*3.99*
hsa-mir-323	*0.53*	*3.64*
hsa-mir-658	*0.04*	*9.09*
hsa-mir-511-2	*0.66*	*2.23*
hsa-mir-379	*0.71*	*1.58*
hsa-mir-92a-1	*0.10*	*-6.37*
hsa-mir-2861	*0.01*	*35.53*
hsa-mir-187	*0.03*	*26.07*
hsa-mir-128-1	*0.58*	*5.09*
hsa-mir-1976	*0.03*	*11.13*
hsa-mir-1262	*0.44*	*5.57*
hsa-mir-422a	*0.57*	*3.26*
hsa-mir-1271	*0.62*	*-2.57*
hsa-mir-433	*0.07*	*-10.33*
hsa-mir-224	*0.60*	*4.83*
hsa-mir-3615	*0.44*	*2.85*
hsa-mir-154	*0.69*	*1.52*
hsa-mir-1-1	*0.50*	*-2.40*
hsa-mir-3127	*0.42*	*4.57*
hsa-mir-3182	*0.57*	*3.19*
hsa-mir-3677	*0.53*	*3.08*
hsa-mir-1283-2	*0.72*	*2.50*
hsa-mir-3605	*0.65*	*2.16*
hsa-mir-1287	*0.66*	*1.57*
hsa-mir-642b	*0.78*	*1.76*
hsa-mir-873	*0.08*	*9.22*
hsa-mir-548aa-1	*0.00*	*4.50*
hsa-mir-548d-1	*0.00*	*4.50*
hsa-mir-184	*0.02*	*8.35*
hsa-mir-504	*0.70*	*-1.37*
hsa-mir-641	*0.65*	*-1.92*
hsa-mir-758	*0.45*	*-2.70*
hsa-mir-3150	*0.47*	*3.77*
hsa-mir-3913-2	*0.41*	*-3.24*
hsa-mir-566	*0.04*	*15.98*
hsa-mir-940	*0.49*	*5.58*
hsa-mir-3651	*0.58*	*4.98*

hsa-mir-2277	*0.60*	*2.95*
hsa-mir-412	*0.59*	*2.22*
hsa-mir-3934	*0.80*	*1.53*
hsa-mir-3940	*0.84*	*1.39*
hsa-mir-550a-2	*0.58*	*1.59*
hsa-mir-550b-2	*0.58*	*1.59*
hsa-mir-580	*0.05*	*55.05*
hsa-mir-939	*0.75*	*18.41*
hsa-mir-1291	*0.54*	*6.49*
hsa-mir-1193	*0.55*	*3.93*
hsa-mir-2355	*0.43*	*3.08*
hsa-mir-1233-2	*0.57*	*2.82*
hsa-mir-18b	*0.69*	*1.53*
hsa-mir-627	*0.72*	*3.17*
hsa-mir-2110	*1.00*	*-1.01*
hsa-mir-662	*0.60*	*-1.93*
hsa-mir-1266	*0.55*	*-2.34*
hsa-mir-668	*0.53*	*-2.57*
hsa-mir-124-2	*0.48*	*-4.00*
hsa-mir-3158-1	*0.90*	*-1.17*
hsa-mir-3158-2	*0.90*	*-1.17*
hsa-mir-1913	*0.03*	*60.62*
hsa-mir-3928	*0.01*	*17.32*
hsa-mir-2116	*0.62*	*5.71*
hsa-mir-1468	*0.85*	*1.36*
hsa-mir-655	*0.98*	*-1.04*
hsa-mir-3912	*0.96*	*-1.08*
hsa-mir-578	*0.62*	*-1.98*
hsa-mir-657	*0.59*	*-4.25*
hsa-mir-133b	*0.09*	*-8.60*
hsa-mir-3622a	*0.58*	*3.52*
hsa-mir-572	*0.02*	*19.29*
hsa-mir-1909	*0.69*	*10.12*
hsa-mir-219-2	*0.73*	*9.13*
hsa-mir-3922	*0.69*	*8.47*
hsa-mir-937	*0.61*	*8.37*
hsa-mir-4298	*0.47*	*8.17*
hsa-mir-3619	*0.71*	*6.67*
hsa-mir-659	*0.74*	*4.85*
hsa-mir-1254	*0.45*	*4.61*
hsa-mir-1288	*0.64*	*4.02*
hsa-mir-624	*0.59*	*2.77*
hsa-mir-31	*0.53*	*2.70*
hsa-mir-3662	*0.83*	*1.46*
hsa-mir-491	*0.85*	*1.36*
hsa-mir-1306	*0.98*	*1.03*

hsa-mir-656	*0.76*	*-1.43*
hsa-mir-877	*0.75*	*-1.47*
hsa-mir-548c	*0.78*	*1.31*
hsa-mir-548z	*0.78*	*1.31*
hsa-mir-1286	*0.60*	*9.64*
hsa-mir-618	*0.56*	*4.03*
hsa-mir-323b	*0.67*	*2.93*
hsa-mir-509-3	*0.71*	*2.76*
hsa-mir-616	*0.85*	*1.40*
hsa-mir-487a	*0.12*	*-8.93*
hsa-mir-3116-2	*0.74*	*5.13*
hsa-mir-539	*0.74*	*5.36*
hsa-mir-506	*0.56*	*3.22*
hsa-mir-3192	*0.72*	*2.62*
hsa-mir-3170	*0.85*	*1.35*
hsa-mir-612	*0.88*	*1.25*
hsa-mir-637	*0.81*	*10.04*
hsa-mir-219-1	*0.68*	*7.15*
hsa-mir-2113	*0.66*	*6.25*
hsa-mir-615	*0.62*	*4.73*
hsa-mir-1299	*0.57*	*4.39*
hsa-mir-1183	*0.71*	*3.42*
hsa-mir-760	*0.71*	*2.53*
hsa-mir-1269	*0.56*	*2.31*
hsa-mir-494	*0.74*	*2.05*
hsa-mir-548w	*0.78*	*1.69*
hsa-mir-548b	*0.78*	*1.62*
hsa-mir-606	*0.84*	*1.43*
hsa-mir-103-2-as	*0.57*	*4.27*
hsa-mir-3913-1	*0.33*	*-3.24*
hsa-mir-3123	*0.82*	*11.11*
hsa-mir-3133	*0.71*	*10.65*
hsa-mir-3939	*0.72*	*9.42*
hsa-mir-554	*0.66*	*8.55*
hsa-mir-1228	*0.67*	*5.87*
hsa-mir-320d-2	*0.75*	*5.60*
hsa-mir-3175	*0.74*	*5.36*
hsa-mir-591	*0.74*	*5.20*
hsa-mir-3139	*0.74*	*5.02*
hsa-mir-4290	*0.74*	*4.85*
hsa-mir-3194	*0.74*	*4.84*
hsa-mir-320e	*0.73*	*4.79*
hsa-mir-3675	*0.57*	*4.58*
hsa-mir-541	*0.57*	*4.39*
hsa-mir-570	*0.73*	*4.28*
hsa-mir-3125	*0.72*	*4.07*

hsa-mir-1915	*0.71*	*3.45*
hsa-mir-515-2	*0.72*	*2.93*
hsa-mir-556	*0.72*	*2.80*
hsa-mir-1289-2	*0.76*	*1.93*
hsa-mir-1282	*0.76*	*1.87*
hsa-mir-548v	*0.77*	*1.71*
hsa-mir-34b	*0.87*	*1.26*
hsa-mir-3691	*0.87*	*1.26*
hsa-mir-1200	*0.89*	*1.22*
hsa-mir-3925	*0.08*	*-7.01*
hsa-mir-650	*0.75*	*14.39*
hsa-mir-4269	*0.66*	*8.55*
hsa-mir-29b-2	*0.78*	*7.35*
hsa-mir-4285	*0.77*	*6.96*
hsa-mir-3176	*0.62*	*6.32*
hsa-mir-3143	*0.75*	*5.60*
hsa-mir-1224	*0.75*	*5.60*
hsa-mir-196a-2	*0.74*	*4.84*
hsa-mir-3617	*0.73*	*4.62*
hsa-mir-3661	*0.57*	*4.34*
hsa-mir-1911	*0.72*	*3.48*
hsa-mir-3144	*0.72*	*2.80*
hsa-mir-3664	*0.72*	*2.80*
hsa-mir-1537	*0.72*	*2.68*
hsa-mir-936	*0.72*	*2.56*
hsa-mir-888	*0.90*	*1.23*
hsa-mir-3138	*0.90*	*1.23*
hsa-mir-1203	*0.90*	*1.23*
hsa-mir-217	*0.43*	*-15.09*
hsa-mir-548q	*0.46*	*-17.45*
hsa-mir-4323	*0.78*	*7.60*
hsa-mir-3161	*0.77*	*6.96*
hsa-mir-3943	*0.77*	*6.96*
hsa-mir-3187	*0.75*	*5.60*
hsa-mir-3153	*0.75*	*5.60*
hsa-mir-516a-2	*0.75*	*5.60*
hsa-mir-921	*0.74*	*4.85*
hsa-mir-216b	*0.73*	*4.28*
hsa-mir-3611	*0.73*	*4.28*
hsa-mir-378b	*0.72*	*3.86*
hsa-mir-4260	*0.73*	*2.46*
hsa-mir-611	*0.73*	*2.46*
hsa-mir-3156-2	*0.73*	*2.46*
hsa-mir-1293	*0.76*	*1.88*
hsa-mir-3167	*0.76*	*1.88*
hsa-mir-661	*0.76*	*1.88*
